# Bidirectional screening and testing for TB and COVID-19 among outpatient department attendees: outcome of an initial intervention in Ghana

**DOI:** 10.1186/s12879-023-08208-w

**Published:** 2023-04-17

**Authors:** Yaw Adusi-Poku, Zeleke Alebachew Wagaw, Rita Patricia Frimpong-Mansoh, Isaac Opoku Asamoah, Felix Sorvor, Felix Kwami Afutu, Charity Sarpong, Luiz Octaviano Amoussou-Gohoungo, Farida Ngelba Abdulai, Sevim Ahmedov

**Affiliations:** 1 National Tuberculosis Programme, Ghana Health Service, Accra, Ghana; 2 Sustaining Technical and Analytical Resources (STAR)/USAID, Accra, Ghana; 3 Regional Health Directorate Greater Accra, Ghana Health Service, Accra, Ghana; 4 United States Agency for International Development (USAID), Bureau for Global Health, Washington, USA

**Keywords:** Simultaneous testing of COVID-19 and TB, Screening of facility attendees, Yield of TB and COVID-19 screening, TB case detection, Ghana

## Abstract

**Background:**

Tuberculosis (TB) remains a major public health threat in Ghana. The impact of COVID-19 resulted in a 15% decline of TB case notification in 2020 compared to 2019. To mitigate the impact on TB services, the Ghana National Tuberculosis Programme (NTP) introduced the bidirectional screening and testing for TB and COVID-19 in 2021.

**Objective:**

To evaluate the yield of bidirectional screening and testing for TB and COVID-19 among facility attendees in the Greater Accra region.

**Method:**

We used secondary data obtained from the initial implementation stage of the bidirectional testing for TB and COVID-19 among COVID-19 and/or TB presumed cases in five health facilities in the Greater Accra Region from January to March 2021. To mitigate the impact of COVID-19 on TB services and accelerate TB case detection, the NTP of Ghana introduced bidirectional screening and testing for TB and COVID-19 in Greater Accra Region before scaling up at national level.

**Results:**

A total of 208 presumed TB or COVID-19 cases were identified: 113 were tested for COVID-19 only, and 94 were tested for both TB and COVID-19, 1 was tested for TB only. Among presumed cases tested for COVID-19, 9.7% (95% CI, 5.6-13.7%) were tested positive. Whilst among the total presumed tested for TB, 13.7% (95% CI, 6.8-20.6%) were confirmed to have TB. Among the total 94 presumed cases tested for both TB and COVID-19, 11.7% (95% CI, 5.2-18.2%) were confirmed to have TB and 13.8% (95% CI, 6.9-20.8%) participants were COVID-19 positive and one participant (1.1%) had both COVID-19 and TB.

**Conclusion:**

Bidirectional screening and testing for TB and COVID-19 shows significant potential for improving overall case detection for the two diseases. The bidirectional screening and testing could be applicable to address a similar respiratory epidemic in the future that might have a masking effect on the response to TB disease.

**Supplementary Information:**

The online version contains supplementary material available at 10.1186/s12879-023-08208-w.

## Introduction

Before the COVID-19 pandemic, TB remained the leading cause of death among infectious diseases globally [1]. Based on the 2021 Global TB report in Ghana, estimated 44,000 people fell ill with TB, and estimated 14,900 people died of TB in 2020 [2]. Of those total estimated incident TB cases in Ghana from 2015 to 2020, only 29–34% were detected and notified to the national tuberculosis programme (NTP) [3]. This implies 66–71% of incident TB cases are missed annually. Among others, one of the driving factors contributing to the low TB case detection rate is low access to TB diagnostics at the lower-level facilities, where most Outpatient Department (OPD) attendees seek first care. The 2017 Ghana patient pathway analysis report indicated that more than 75% of patients seek care first at facilities with limited TB diagnostic capacity [4]. To increase access to TB testing, the NTP introduced a sputum sample transportation system in October 2019 in 1000 peripheral facilities and linked them to 126 GeneXpert testing sites [5]. Although the sputum sample transport was introduced and implemented only in the last quarter of 2019; the programme notified an additional 700 new cases in 2019 as compared to the 2018 report [5]. However, the progress made has been halted after the emergence of COVID-19. As a result, the TB case notification declined by 15% in 2020 compared to the 2019 achievement [6].

A month after World Health Organization (WHO) declared COVID-19 as a public health emergency of international concern on 30 January 2020, [7]. Ghana reported its first two COVID-19 cases on 12 March 2020 [8]. Following the crisis of COVID-19, Ghana implemented a partial lockdown followed by a full-scale country lockdown. Consequently, the number of clients visiting health facilities declined drastically (up to 30%), [6] and the total number of TB cases notified in Ghana in 2020 (12,674) [3] declined by 15% as compared to the 2019 notification (14,691) [3]. Globally, in 2020, the number of people newly diagnosed with TB was declined by 18% [1]. Though the decline in case notification seen in Ghana is comparable with the global average, it is much higher than the WHO African region (2.5%) [1]. The decline in the number of cases notified might be linked to double stigma against TB and COVID-19 symptoms and shifting the health service focus to COVID-19 [9]. In 2020, health workers, testing machines, laboratories, and health facilities were diverted from exiting TB service to COVID-19 response [9]. With similar signs and symptoms such as cough, fever, and difficulty breathing, TB and COVID can confuse people, and people might not need to go to health facilities because of fear and stigma [9]. Though COVID-19 impacted TB globally, integrating the COVID-19 and TB services might be a conduit for new opportunities and lessons to end the TB epidemic. Ghana’s National Tuberculosis Programme (NTP) is implementing new innovative interventions to restore and accelerate TB services in the country to achieve the country targets set based on the United Nations’ sustainable development goal and WHO end TB strategy targets. The NTP introduced bidirectional screening and testing for COVID-19 and TB in Ghana in 2021. Bidirectional screening and testing for TB and COVID-19 refers to simultaneous screening and testing for both TB and COVID-19. This intervention was expected to bring synergy in finding both COVID-19 and TB cases as they share similar signs and symptoms. The bidirectional screening was implemented using the TB and/or COVID-19 symptom screening tools among all OPD attendees in each implementing facility. After the symptom screening, those eligible “presumed to have TB or COVID-19) were tested for COVID-19 and TB using Cepheid’s GeneXpert® as one and same platform, in Accra from January to March 2021.

## Objective

The study aims to evaluate the yield of testing for TB and COVID-19 among presumed TB or CVID-19 cases identified at the initial implementation stage of the bidirectional screening and testing for the two diseases.

## Method

The study was conducted using secondary data obtained from five health facilities implementing bidirectional screening and testing of COVID-19 and TB in the Greater Accra Region from January to March 2021. During the COVID-19 pandemic, TB service was affected in various ways, including a shift of focus to COVID-19; a reduced index of suspicion to TB by health providers, and in most facilities, there was a task shifting from tuberculosis case finding to COVID-19. Knowing that the symptoms and signs of TB and COVID-19 are similar and the reduced index of suspicion to TB during the COVID-19 pandemic, the National TB Control Programme developed a policy for bidirectional screening and testing for TB and COVID-19. Following the policy, the programme introduced bidirectional screening and testing for TB and COVID-19 in five facilities in the Greater Accra Region before the programme mobilised resources and scaled up the intervention at the national level. The five facilities that started the implementation of the initial stage of the intervention and included in our study were: Achimota Hospital, Tema General Hospital, Ridge Hospital, Ga West Municipal Hospital and Madina Policlinic. COVID-19 tests for Madina Polyclinic were conducted at Noguchi Memorial Institute for Medical Research (NMIMR). Record on bidirectional testing was retrieved using a predesigned excel data extraction form. Retrieved data include age, sex, symptoms, TB treatment history, TB test results, and COVID-19 results.

### Patient screening

**Fig. 1:**
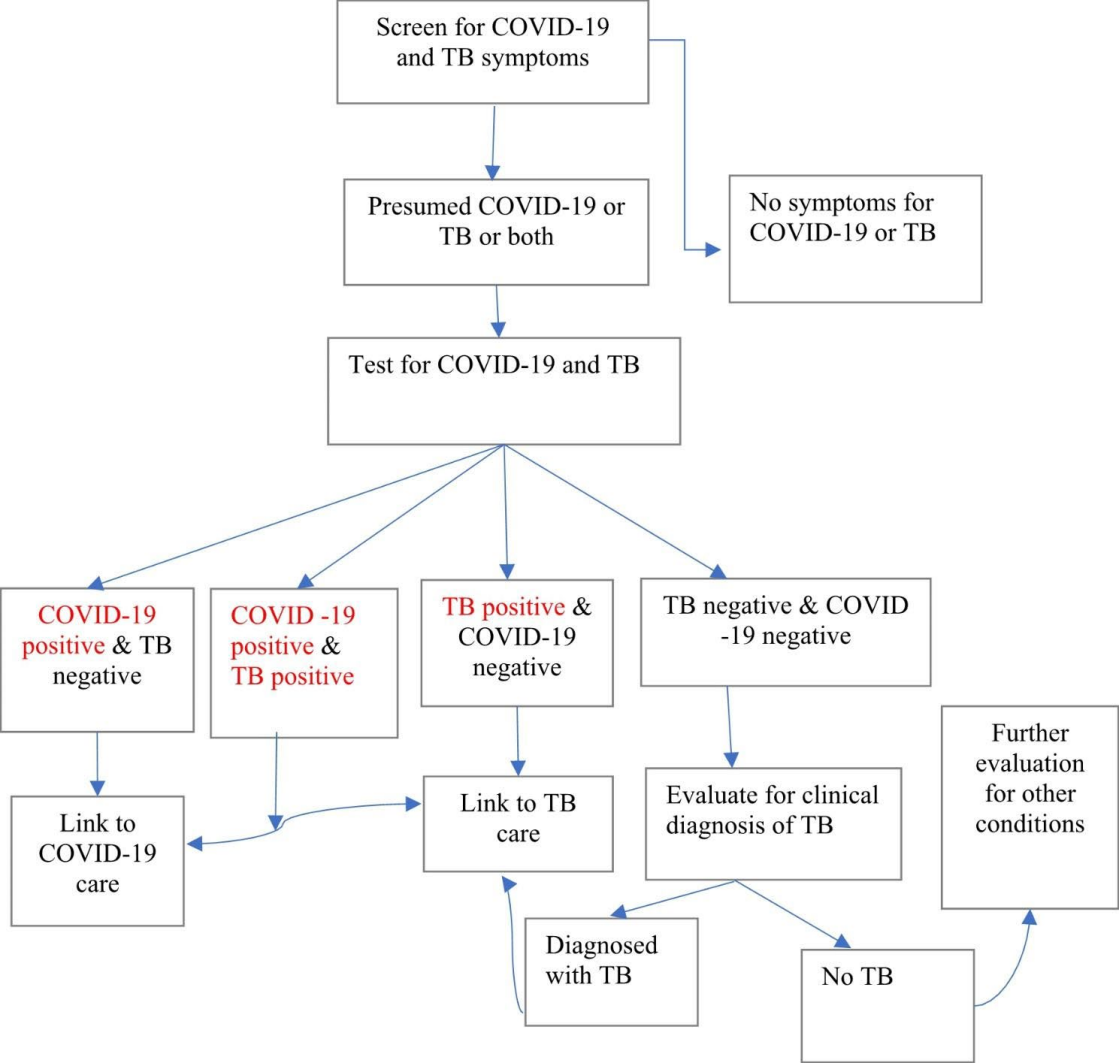
Algorithm for bidirectional screening and testing of COVID-19 and TB among outpatient attendees in Ghana. (source- Ghana NTP)

Following the decision made by the NTP of Ghana on the implementation of bidirectional screening for TB and COVID-19, triage nurses or trained task-shifting officers screened patients for TB and/or COVID-19 symptoms at the outpatient department (OPD) as part of the routine service. The screening was conducted based on the TB and COVID screening algorithms. The screening symptoms include cough, night sweats, weight loss, fever, chest pain and other symptoms like headache and muscle pain as reported by clients. Clients with any symptoms of TB and/or COVID-19 were considered eligible for both TB and COVID-19 tests. To increase the index of suspicion for the two diseases, the program offered tests for all presumed to have COVID-19 and/or TB. This includes clients with COVID-19 symptoms but not relevant TB like loss of test. But it excluded COVID contacts without any symptoms or people who referred themselves to COVID-19 tests without symptoms. The bidirectional screening and testing were conducted based on the algorithms presented in Fig. 1.

### Specimen collection and laboratory tests

Naso-pharyngal/Oro-pharyngal swab and sputum samples were collected for each client for COVID-19 and TB tests, respectively. Both TB and COVID-19 tests were conducted using molecular PCR (Xpert ® MTB*/*RIF and Xpert Xpress SARS-CoV-2). Cepheid’s Xpert Xpress SARS-CoV-2 cartridge was used for the qualitative detection of COVID-19 and Xpert ® MTB*/*RIF cartridge to detect mycobacterium tuberculosis and rifampicin resistance. Both TB and COVID testes were performed based on the manufacturer’s instructions.

### Data entry and analysis

Using a data abstraction form (excel sheet), we retrieved data at the five health facilities where bidirectional TB and COVID-19 screening, and testing were being implemented for the first time. Data were collected to document the initial experience of bidirectional screening before full scale up of the interventions at the national level. After data cleaning, the retrieved excel data was exported and analysed using Stata release V17 (StataCorp, College Station, Texas, USA, 2021). We computed the percentages with 95% confidence interval of COVID-19 and TB positive among participants to determine the prevalence of each disease among the total screened and tested.

### Ethical consideration

This study was conducted based on the secondary data of the routine services, which is public data. No additional data was collected for the purpose of this study. The study maintained the confidentiality of the information of each patient. The patient’s name was not included in the data abstraction form and study dataset. We used patient ID and the name of a facility together to identify participants.

## Results

The five facilities included in the study documented 208 patients with respiratory symptoms between January 2021 and March 2021. Among 206 presumed TB and/or COVID-19 cases with sex information available, males accounted for 104 (50.5%). The median age for participants was 40 years, with minimum and maximum age of 9 and 96 years, respectively (Table 1). The most common symptoms reported by presumed cases include cough in 192 (92.3%), fever in 24 (11.5%), and weight loss in 8 (3.8%) of participants. A total of 10 (4.8%) presumed cases reported that they had been treated for TB in the past (Table 1).

All 208 presumed cases had at least one test result: 94 (5.2) were tested for both TB and COVID-19; 113 (54.3%) were tested only for COVID-19 and one participant (0.5%) was tested only for TB.

**Fig. 2:**
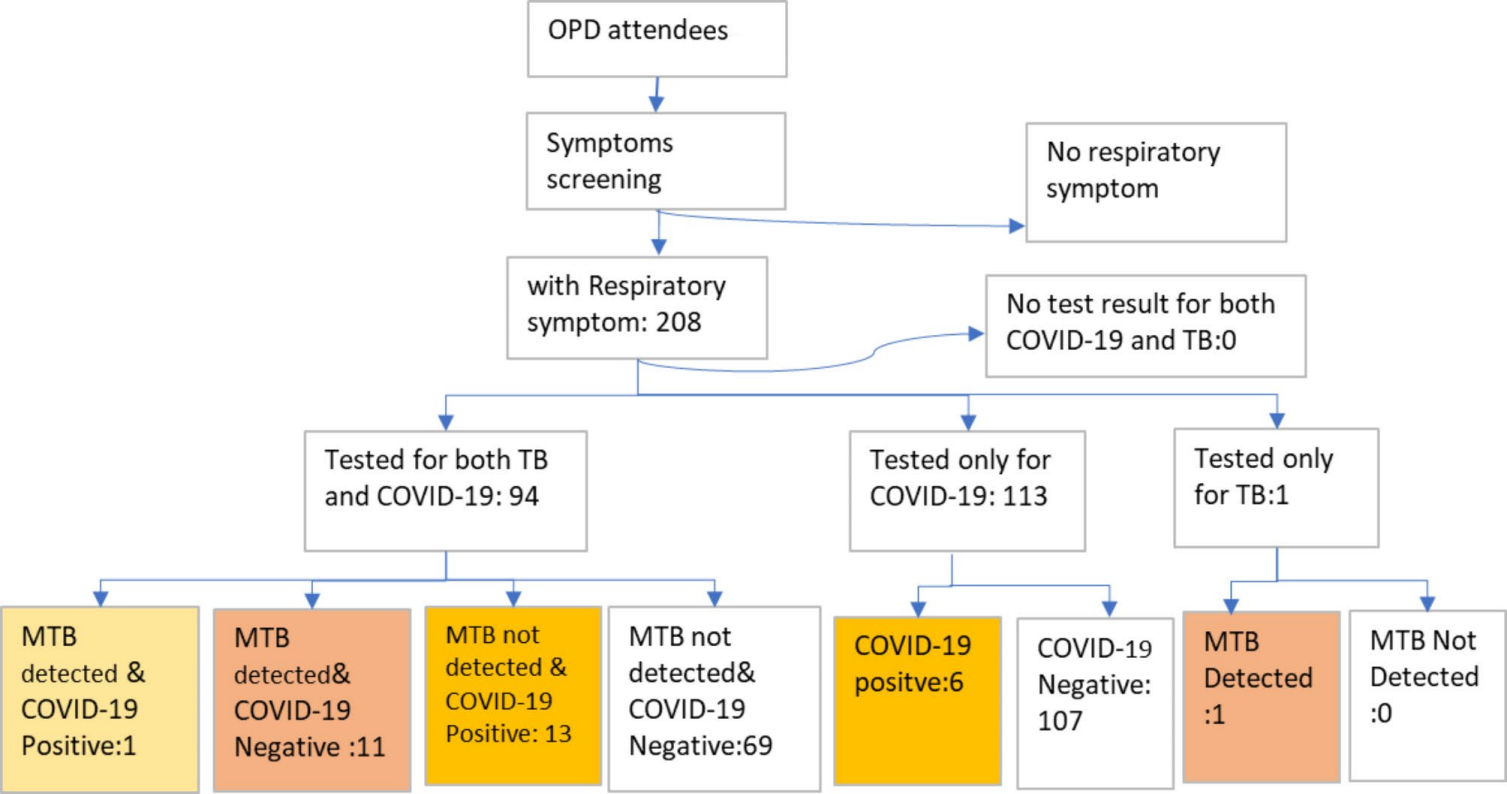
The result of bidirectional screening and testing for TB and COVID-19 among five health facilities’ attendees in the Greater Accra Region from January to March 2021

A total of 113 (54.3) presumed TB cases did not submit sputum samples for TB test because of the inability to produce sputum or patient did not go to the laboratory as reported by the service providers (Fig. 2). Among the total presumed, a total of 19 (9.1%) COVID-19 positives, 12 (5.8%) MTB positive and one (0.5%) both MTB and COVID-19 positive cases were confirmed. (Fig. 2). All detected TB cases were rifampicin sensitive.

### Prevalence of bacteriologically confirmed TB among presumed cases tested

Among the 95 presumed cases tested for TB, 13 (13.7% (95% CI, 6.8-20.6%)) were confirmed to have TB. Among 58 males tested for TB, 10 (17.2% (95% CI,7.5-27%)) were confirmed to have TB, whilst among the total 38 females tested for TB, 3 (8.1%) were confirmed to have TB (Table 1).

### Prevalence of COVID-19 among presumed cases tested

Among the 207 presumed cases tested, 20 (9.7% (95% CI, 5.6-13.7%)) tested positive for COVID-19. Among the 103 presumed males tested, 14 (13.6% (95% CI, 7.0-20.2%)) were confirmed to have COVID-19, and among 102 presumed females tested, 6 (5.9% (95% CI, 1.3-10.4%)) were confirmed to have COVID-19 (Table 1).

### Prevalence of COVID-19 and TB among presumed cases tested for both TB and COVID-19

Of the 94 tested for both TB and COVID-19, 25 (26.6% (95% CI, 17.7-35.5%)) had at least one positive result for TB or COVID-19: (1) 11 participants (11.7% (95% CI, 5.2-18.2%)) had MTB positive and COVID-19 negative result, (2), one participant (1.1%) had both TB and COVID-19 Positive results (3) 13 participants (13.8% (95% CI, 6.9–20.8)) had COVID 19 Positive and MTB negative results (Table 1).

**Table 1:** Bidirectional screening and testing for COVID-19 and TB results among outpatient department attendees in five health facilities in the Greater Accra Region, January to March 2021

Variable	Frequency	%	95% CI
**Total presumed to have COVID or TB**	208	100	
**sex, n = 206**			
Male	104	50.5	
Female	102	49.5	
**Age, n = 203, Median age = 40**			
10–14	7	3.4	
15–24	23	11.3	
25–34	45	22.2	
35–44	49	24.1	
45–54	40	19.7	
55–64	18	8.9	
65+	21	10.3	
**Treated for TB before? n = 208**			
Yes	10	4.8	(1.9, 7.7)
**Symptoms reported, n = 208**			
Cough	192	92.3	(88.7, 95.9)
Fever	24	11.5	(7.2, 15.9)
Weight loss	8	3.8	(1.2, 6.5)
Other symptoms	54	26	(20, 31.9)
**COVID-19 test result, n = 207**			
Positive	20	9.7	(5.6, 13.7)
**COVID-19 result by sex**			
Male, n = 103			
Positive	14	13.6	(7.0, 20.2)
Female, n = 102			
Positive	6	5.9	(1.3–10.4)
**MTB test result, n = 95**			
Positive (MTB detected)	13	13.7	(6.8, 20.6)
**MTB result by sex**			
Male, n = 58			
Positive	10	17.2	(7.5, 27)
Female, n = 37			
Positive	3	8.1	(0, 16.9)
**Teste result for those tested for both COVID and TB, n = 94**			
COVID-19 and/or TB positive	25	26.6	(17.7,35.5)
TB Positive COVID Negative	11	11.7	(5.2,18.2)
TB Positive and COVID Positive	1	1.1	
COVID-19 Positive-TB Negative	13	13.8	(6.8,20.8)
Negative for both TB and COVID-19	69	73.4	(64.5,82.3)

## Discussion

Though COVID-19 was the leading cause of death among infectious diseases globally in 2020, in Ghana, TB is still the leading cause of death above COVID-19: 14,900 estimated TB mortality in 2020 ^2^ versus 333 reported COVID-19 deaths [10] in the same year. In 2020 both numbers screened for TB and TB cases notified declined as a result of the impact of COVID-19 [6]. To mitigate the impact of COVID-19 on TB service, the NTP of Ghana started the implementation of bidirectional screening and testing of COIVD-19 and TB among facility attendees in the Greater Accra Region. Based on the experience observed in the five facilities, the programme scaled up the intervention in all COVID-19 and TB testing sites all over the country in the third quarter of 2021 (unpublished). After the scaling up of the intervention the TB case notification rate increased by 4% in 2021 compared to 2020 [11].

Both COVID-19 and TB are airborne diseases and manifest similar signs and symptoms such as cough, fever and breathing difficulty [12]. The decision for simultaneous testing of both TB and COVID-19 for the same patient depends on the local epidemiology of the two diseases, clinical future and individual risk factors of a patient [12]. The total number of COVID-19 positives in the study area (Greater Accra Region) as at February 19, 2023, was 97,242, while the total COVID-19 positive cases at the national level, excluding international travelers, was 163,477 [13]. Greater Accra contributed 69% of the total COVID-19 cases detected at the national level [13]. During the study period (January to March 2021), a total of 35,812 new COVID-19 cases were reported at the national level, [14] equivalent to 21% of the total cumulative cases notified in the country as at February 19, 2023.

This bidirectional screening and testing of the two diseases yielded a higher proportion of TB cases (3.7% point increased) and lower proportion of COVID-19 cases (2% point decreased) as compared to the yield of a single disease screening strategy at its respective screening unit of COVID-19 or TB during the study period. The test positive rate for TB and COVID-19 in the routine service at the national level during the study period was 10% for TB ( NTP report, unpublished) and 12% for COVID-19 ^14^.

The yield of TB among presumed cases in our study (13.7%) is higher than the finding of Afum T et al., which reported 0.8% TB cases among COVID-19 presumed cases in Accra in 2021 [15]. The yield of COVID-19 among presumed cases in our study (9.7%) is slightly less than the finding of Afum T et al., which reported SARS COV-2 infection of 14.7%.^15^ One possible reason for the observed variation in the yield of the two diseases between our study and the second study is a variation in the general characteristics of patients screened for the two diseases. In our study, the study participants were patients at the outpatient department. In contrast, the study participant in the second study is the COVID-19 presumed cases refereed from different facilities for COVID-19 testing using the routine COVID-19 surveillance activity.

The prevalence of both TB and COVID-19 were higher in males than in females. This finding is consistent with the finding of Afum T et al [15]. The 2013 Ghana TB prevalence survey result also revealed a higher prevalence of TB among males than females [16].

Regarding to TB and COVID-19 comorbidities, among the total confirmed 14 COID-19 cases who had TB test result, one patient (7.1%) had also TB. A meta-analysis study done in 2021 reported a pooled estimate of 1.07% pulmonary TB among COVID-19 cases, with a range of 0.8–14.2% [17]. However, there is variation in the study participants in the two studies: in our study participants were presumed cases of TB or COVID-19 who were evaluated for both TB and COVID-19 at the same time whereas the participants for the second study were CVID-19 cases who were assessed later for TB.

This study finding has revealed that implementing screening and testing of only one disease (TB or COVID-19) may lead to missing a significant number of cases for the second one. This study supports the recommendation of WHO, STOP TB Partnership, the Global Fund and USAID of implementing simultaneous testing of TB and COVID-19 to improve case detection for the two diseases [18]. In a country like Ghana, where the prevalence of TB is high in the community, [16] implementing the COVID-19 Response Programme alone may interfere with TB diagnosis. The bidirectional screening and testing of COVID − 19 and TB will leverage the synergy of the two programmes and save resources as the same health worker and the same testing platform is used. However, the low testing rate for TB among presumed cases (95/208) is a concern. This is consistent with the routine intensified TB cases finding annual performance report 2015–2019 (unpublished), which reported that there was a 50% testing rate among presumed cases identified.

This study has the following major limitations: the study lacks information on the total number of people screened for COVID or TB. Facilities recorded only presumed COVID-19 and/or TB cases. The study also lacks information on the reason why some participants were not tested for TB.

## Conclusion

Bidirectional screening and testing of COVID-19 and TB among presumed clients at health facilities yielded a significant proportion of COVID-19 and TB cases. The initial implementation experience in Greater Accra Region suggests the need for broader implementation. However, there is a need to resolve implementation challenges, particularly the low testing rate for TB among presumed cases. Capacity building for health workers on sputum production should be part of the ongoing activities. Further study needs to be done to explore factors affecting testing rates for TB among presumed. The bidirectional screening and testing might also be applicable for a similar respiratory epidemic in nature in the future that may mask the efforts in fighting against the TB disease. It should also be emphasised that there is a need for an update on the decision on dual screening based on the present local epidemiological evidence and over time.

## Electronic supplementary material

Below is the link to the electronic supplementary material.

Supplementary Material 1

## Data Availability

The dataset used/analysed is available from principal investigator and corresponding author on reasonable request. Currently, the data is available in google cloud at the following link: TB-COVID Test Data Accra 2021 .

## Abbreviations

CI: Confidence Interval
COVID-19: Corona Virus Disease 19
MTB: Mycobacterium Tuberculosis
NTP: National Tuberculosis Programme
OPD: Outpatient Department
TB: Tuberculosis
USAID: United States Agency for International Development
WHO: World health organization
